# Wound Healing with Medications for Rheumatoid Arthritis in Hand Surgery

**DOI:** 10.5402/2012/251962

**Published:** 2012-12-03

**Authors:** A. R. Barnard, M. Regan, F. D. Burke, K. C. Chung, E. F. S. Wilgis

**Affiliations:** ^1^Pulvertaft Hand Centre, Kings Treatment Centre, Royal Derby Hospital, Uttoxeter Road, Derby DE22 3NE, UK; ^2^Department of Rheumatology, Royal Derby Hospital, Uttoxeter Road, Derby DE22 3NE, UK; ^3^A. Alfred Taubman Health Care Center, University of Michigan Health System, 1500 East Medical Center Drive, Ann Arbor, MI 48109, USA; ^4^Curtis National Hand Center, Union Memorial Hospital, Johnston Professional Building, 2nd Floor, 3333 North Calvert Street, Baltimore, MD 21218, USA

## Abstract

*Introduction*. Medications used to treat rheumatoid arthritis, such as corticosteroids, disease-modifying agents (DMARDs), and injectable biological agents (anti-TNF**α**), may have widespread effects on wound healing. In hand surgery, it is important to balance the risks of poor wound healing from continuing a medication against the risks of a flare of rheumatoid arthritis if a drug is temporarily discontinued. *Materials and Methods*. A United Kingdom (UK) group of 28 patients had metacarpophalangeal joint replacement surgery in 35 hands (140 wounds). All medication for rheumatoid arthritis was continued perioperatively, except for the injectable biological agents. *Results*. There were no instances of wound dehiscence or deep infection and only one episode of minor superficial infection. *Conclusions*. We conclude that provided care is taken to identify and treat any problems promptly, it is appropriate to continue most antirheumatoid medications in the perioperative period during hand surgery to reduce the risk of destabilising the patients' overall rheumatoid disease control.

## 1. Introduction

Hand deformities are common in rheumatoid arthritis (RA), with 45% of patients affected by ulnar drift at the metacarpophalangeal joints (MCPJs) after 5 years of disease activity and with 33% no longer able to work [1]. Surgery is often performed to improve hand function and relieve pain resulting from tendon and joint degeneration. A common procedure is prosthetic metacarpophalangeal joint (MCPJ) replacement for the treatment of ulnar drift and MCPJ subluxation. Many patients are taking a combination of medications, such as analgesia or anti-inflammatory agents in addition to one or more disease-modifying antirheumatic drugs (DMARDs) to manage their systemic disease [2]. 

There are well-documented concerns in the general literature regarding the use of nonsteroidal anti-inflammatory drugs (NSAIDs), steroids, and immunomodulatory medications in the perioperative period, particularly with respect to wound healing problems. However, specific data pertinent to patients with rheumatoid arthritis is relatively sparse, and recommendations are frequently based upon data from other patient groups, such as in transplant surgery and the management of inflammatory bowel disease [3]. There are studies assessing postoperative complications with orthopaedic surgery in patients with RA, but with the exception of methotrexate, the studies are usually small and retrospective [4]. In cases where prosthetic devices are inserted, a wound breakdown or infection can be devastating and potentially lead to the loss of an implant. However, these concerns must be carefully balanced against the need to maintain disease stability, as a flare of rheumatoid disease activity in the postoperative period may hamper rehabilitation and impair the final outcome.

The effects of anti-inflammatory and immunomodulatory medications on the different stages of the wound healing process can be widespread. The initial inflammatory phase may be blunted, or the proliferative and remodelling phases may be abnormal or prolonged [5].

This retrospective analysis of patients with rheumatoid arthritis presenting for metacarpophalangeal joint (MCPJ) replacement surgery looks at the rate of postoperative complications affecting 140 wounds in 35 hands when the majority of medications were continued.

## 2. Materials and Methods

An existing database of patients with rheumatoid arthritis was used. All have undergone Swanson's metacarpophalangeal joint (MCPJ) arthroplasties under the care of one surgeon at the Pulvertaft Hand Centre. The database is part of a multicentre UK/USA prospective trial comparing medical and surgical management of MCPJ disease. This paper is restricted to the United Kingdom arm of the study and includes assessment of the database and medical notes for the group that went forward to surgery.

Current medications were documented pre- and postoperatively. Drugs were grouped as nonsteroidal anti-inflammatory drugs (NSAIDs), biological agents, noncytotoxic or cytotoxic disease modifying antirheumatic drugs (DMARDs), steroids, or simple analgesics (see Table 1). Anticoagulant use was also recorded. 

A standard operative technique was used under tourniquet control, with access via four dorsal longitudinal incisions to replace four MCP joints in each hand. Perioperative antibiotic prophylaxis was used, with a nonabsorbable monofilament suture for wound closure. The only medication that was routinely altered in the perioperative period was etanercept, an injectable biological anti-TNF agent. This was stopped 2 or 3 weeks preoperatively and restarted once wounds were healed (usually at 2 weeks).

Routine postoperative care included dressing changes and application of dynamic splints with radial bias at the 5th postoperative day (for daytime use) with a static night splint to control extension lag and ulnar drift. Sutures were removed at 2 weeks, and patients remained under the care of the hand therapists thereafter unless problems arose. Dynamic splints were removed at 8–10 weeks. The night splint was commonly used longer. Patients were reviewed by the doctor again at six months, one, two and three years. At each visit, current medication use was recorded and any complications were noted.

## 3. Results

Twenty eight patients were included in the study, representing 35 hands and 140 wounds in total. Twenty five patients were females (mean age 63 years, range 50–76 years), and three cases were males (mean age 50 years, range 44–55 years). Two patients were regular cigarette smokers (10 per day and 20 per day), and none were being treated for diabetes. The distribution of medication use and complications is shown in Table 1. 

All wounds were reviewed at 2 weeks, and sutures were removed at this stage in 34 of 35 cases. There were no serious complications; however, four patients encountered minor problems in the postoperative period.

### 3.1. Patient 1—Delayed Wound Healing

A 51-year-old female, who was taking a cytotoxic DMARD (methotrexate) and codydramol and smoked 20 cigarettes per day, had slightly delayed wound healing. Half the sutures were left for a total of 3 weeks as the wounds were not considered clinically ready for suture removal in one area at 2 weeks.

### 3.2. Patient 2—Superficial Infection

A 63-year-old female, who was taking a cytotoxic DMARD (methotrexate), an NSAID (diclofenac), and a noncytotoxic DMARD (gold injections), developed clinical signs of an early superficial wound infection within one week of surgery. This settled without dehiscence following a short course of oral flucloxacillin. 

### 3.3. Patient 3—Presumed Rheumatoid Flare

A 63-year-old female had surgery to both hands one year apart. After the first operation, when she was taking a cytotoxic DMARD (azathioprine), a steroid (prednisolone), and high-dose aspirin (300 mg), she developed a red painful area around the replaced 5th MCP joint 4 weeks after surgery. This settled after 3 days of rest, elevation, and oral flucloxacillin. Ten days after the second operation, when she was still taking the cytotoxic DMARD and steroid at the same doses, but a lower dose of aspirin, she developed erythema and pain around her wounds and was admitted for 48 hours of rest, elevation and intravenous flucloxacillin. Thereafter, her recovery was uneventful. Senior clinicians' opinions at the time were that these were episodes of rheumatoid flare rather than infective processes. None of her antirheumatoid medications were altered perioperatively.

### 3.4. Patient 4—Late Suture Granuloma

During long-term followup, a 52-year-old lady who was taking an NSAID (naproxen) and a biological agent (etanercept) developed a suture granuloma at 5 months which required surgical excision under local anaesthesia.

## 4. Discussion

Current UK guidelines generally make only brief reference to the perioperative management of antirheumatoid medical therapy. The issue is not mentioned at all in the current American College of Rheumatology recommendations on RA medications [6]. The traditional stance had been that cytotoxic medications should be stopped a few weeks before surgery and should not be recommenced until wounds had healed. However, in recent years there has been a trend towards continuing medications to reduce the risk of a flare of RA. Whilst there are good data to support the continuation of methotrexate, data for other DMARDs and the biological agents has been sparse [3, 7].

There are many ways in which wound healing may be affected by the medications used in RA. Systemic corticosteroids affect gene expression and thereby may blunt the inflammatory phase of wound healing, reduce the rate of reepithelialisation, and alter remodelling of a wound [5]. However, the doses used in RA are usually low (2.5–5 mg daily in our group), and at these doses there has been no evidence of increased complications following joint replacement [4]. In addition, the risk of hypothalamic-pituitary-adrenal axis suppression with higher steroid doses precludes stopping these medications suddenly, and additional supplementation is usually recommended for major surgery [3].

Methotrexate particularly affects macrophages and T lymphocytes and was shown in animal studies to reduce early wound tensile strength [8], but at the lower doses used in RA, and in combination with folate supplementation, there has been no clinical evidence over the years to support a detrimental effect on wound healing [4]. The effects of the cytotoxic DMARDS leflunomide and azathioprine on wound healing are not clear, but limited animal and clinical data show no clear adverse perioperative effects [3]. Likewise, the data on the noncytotoxic DMARDs sulfasalazine and hydroxychloroquine are limited, but most authors regard the latter as minimally toxic and recommend its continuation [3, 4].

The data assessing anti-TNF agents and wound healing are conflicting and comprise a mixture of small retrospective and prospective studies in RA and a larger study in Crohn's disease. For example, Bibbo and Goldberg [9] prospectively assessed 31 patients with RA who underwent a variety of elective foot and ankle procedures. The groups were split into those receiving an anti-TNF agent and those who were not, with all patients continuing all regular medications. Despite the anti-TNF group having a six times higher rate of smoking, they still exhibited similar wound healing and infection complication rates. Den Broeder et al. [10] conducted a larger cohort study assessing risk factors for infection rates following 1209 varied elective orthopaedic procedures in patients with RA. There were apparently more wound dehiscences (9.8%) in those continuing an anti-TNF agent compared to those who were not taking one (4.4%). Although the differences in infection rates were not statistically significant between those who continued anti-TNF therapy (8.7%), those who stopped it (5.8%), and those who were never taking an anti-TNF agent (4%), it is important to note that only a small proportion of procedures were actually undertaken on patients taking these medications (196/1219). A recent systematic review [7] assessing the risk of orthopaedic surgical site infection in patients with RA taking anti-TNF agents concluded that there was insufficient data internationally to reach any conclusion. The topic of anti-TNF agents is reviewed in detail by Pieringer et al. [3] and is also considered in the guidelines discussed below.

In this study, we continued all medications except the anti-TNF agents. We observed a low perioperative complication rate, with only one episode of minor wound infection (1/35 hands), one episode of minor delay in wound healing (1/35 hands), and one patient (whose medications were not altered) probably affected by flares of RA activity (2/35 hands). There was only one late wound complication of a suture granuloma (1/140 wounds).

The recent British Society for Rheumatology DMARD therapy guidelines suggest that methotrexate is unlikely to increase the risk of infection or surgical complications if continued [2]. This is predominantly influenced by one large prospective randomised controlled trial of methotrexate use in 388 rheumatoid patients undergoing elective orthopaedic procedures, which showed no evidence of increased complications with perioperative continuation of methotrexate [11]. Further data analysis suggested increased infection and complication rates with other antirheumatoid medications; however, these subgroups were smaller, and the trial was designed to assess methotrexate.

The 2005 British Society for Rheumatology (BSR) anti-TNF*α* (Tumour necrosis factor *α*) guidelines base perioperative advice upon drug company recommendations, stating that *“treatment with infliximab, etanercept, and adalimumab should be withheld for 2 to 4 weeks prior to major surgical procedures.”* They recommend restarting treatment when wound healing is satisfactory, and there are no signs of infection [12]. This was supported by the British Society for Rheumatology Biologics Register data [13] which showed a four-fold increase in the rate of skin and soft tissue infections with the use of biological agents in a cohort of 8973 RA patients observed prospectively. The current BSR anti-TNF guidelines [14] are essentially no different, except that more specific recommendations are made regarding the timing of stopping the different anti-TNF agents before surgery, based upon pharmacological data and clinical expertise.

This retrospective study of 140 wounds supports a policy of continuing all medications used for rheumatoid arthritis, except the injectable biological agents, in the perioperative period. Although the numbers are small, they add to the growing body of literature regarding the use of these medications. There is unlikely to be any detrimental effect on wound healing in patients undergoing joint replacement in the hand, provided that constant vigilance in the postoperative period is maintained. Furthermore, this is unlikely to interfere with overall RA disease control.

## Figures and Tables

**Table 1:** Medication groups in 35 hands.

Medication group*	Examples (no. of patients)**	Total no. of hands per group	Medication continued	No. of wound breakdowns	No. of wound infections
NSAIDS	Diclofenac (6), ibuprofen (4), flurbiprofen (1), indomethacin (2), naproxen (6), nabumetone (1)	20	Yes	0	1
Cox-2 Inhibitors	Celecoxib, rofecoxib	0	—	—	—
Steroids	Prednisolone	8	Yes	0	0
Biologics	Etanercept (4)	4	No	0	0
Noncytotoxic DMARDs	Gold (1), hydroxychloroquine (2), sulfasalazine (8)	11	Yes	0	1
Cytotoxic DMARDs	Leflunomide (1), methotrexate (20), azathioprine (3)	24	Yes	0	1
Anticoagulants	Warfarin, heparin	0	—	—	—
Aspirin***	Dose 75–300 mg daily	5	Yes	0	0
No medication		2	—	0	0

Key: NSAIDs: nonsteroidal anti-inflammatory drugs, Cox-2: cyclo-oxygenase 2 inhibitors, DMARDs: disease modifying antirheumatic drugs.

*General analgesics excluded, for example, paracetamol, nefopam, codeine, morphine.

**Some patients were taking more than one medication in a group.

***Aspirin was used for cardiovascular risk reduction (75–300 mg) rather than analgesia/anti-inflammatory.
